# NMR analysis of *t*‐butyl‐catalyzed deuterium exchange at unactivated arene localities

**DOI:** 10.1002/jlcr.3440

**Published:** 2016-09-19

**Authors:** Douglas E. Stack, Rachel Eastman

**Affiliations:** ^1^ University of Nebraska at Omaha Chemistry6001 Dodge StreetOmahaNE68182USA

**Keywords:** arene, estrone, NMR analysis, reversible, t‐butyl catalyzed

## Abstract

Regioselective labelling of arene rings via electrophilic exchange is often dictated by the electronic environment caused by substituents present on the aromatic system. Previously, we observed the presence of a *t*‐butyl group, either covalently bond or added as an external reagent, could impart deuterium exchange to the unactivated, C1‐position of estrone. Here, we provide nuclear magnetic resonance analysis of this exchange in a solvent system composed of 50:50 trifluoroacetic acid and D_2_O with either 2‐*t*‐butylestrone or estrone in the presence of *t*‐butyl alcohol has shed insights into the mechanism of this *t*‐butyl‐catalyzed exchange. Fast exchange of the *t*‐butyl group concurrent with the gradual reduction of the H1 proton signal in both systems suggest a mechanism involving ipso attack of the *t*‐butyl position by deuterium. The reversible addition/elimination of the *t*‐butyl group activates the H1 proton towards exchange by a mechanism of *t*‐butyl incorporation, H1 activation and exchange, followed by eventual *t*‐butyl elimination. Density functional calculations are consistent with the observation of fast *t*‐butyl exchange concurrent with slower H1 exchange. The σ‐complex resulting from ipso attack of deuterium at the *t*‐butyl carbon was 6.6 kcal/mol lower in energy than that of the σ‐complex resulting from deuterium attack at C1. A better understanding of the *t*‐butyl‐catalyzed exchange could help in the design of labelling recipes for other phenolic metabolites.

## 1 Introduction

**Figure 1:**
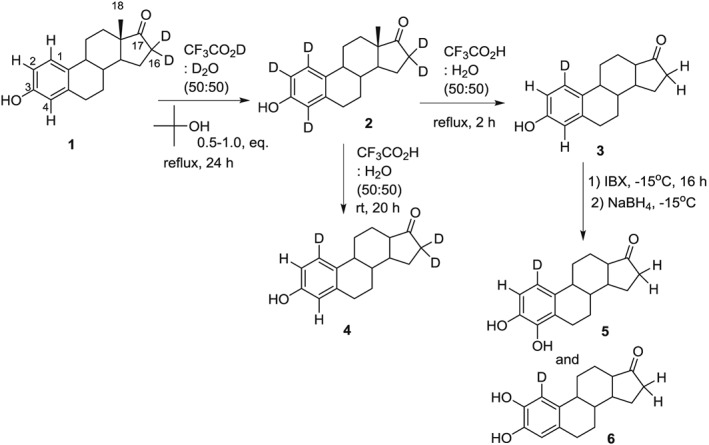
Regioselective labeling of estrone‐1‐d and conversion to labeled catechols

We have recently developed a regioselective method to impart deuterium exchange at a position unactivated by the presence of an electron‐donating group.^1^ In particular, the phenol ring of estrone was labeled at C1 using a novel *t*‐butyl‐catalyzed exchange recipe. Figure 1 shows how this method was used to synthesize estrone‐1‐*d* (**3)** and the catechol estrogen metabolites of estrone, 4‐hydroxyestrone‐1‐d (4‐OHE_1_–1‐*d*) (**5)** and 2‐hydroxyestrone‐1‐d (2‐OHE_1_–1‐*d*) (**6)**. The catechol estrogen metabolites of both estrone (E_1_) and β‐estradiol (E_2_) were further oxidized to genotoxic *o*‐quinones.^2^, ^3^, ^4^ 4‐OHE_1_ and 4‐OHE_2_ are carcinogenic in animal models^5^, ^6^ and have been associated with the occurrence of several different human cancers.^7^, ^8^ When the *o*‐quinone of 4‐OHE_1_ reacts with DNA at either the N3 position of adenine or the N7 position of guanine, it does so at C1.^9^, ^10^ We have recently used compound **5** to investigate the mechanism of DNA modification caused by estrogen *o*‐quinones.^11^

**Figure 2:**
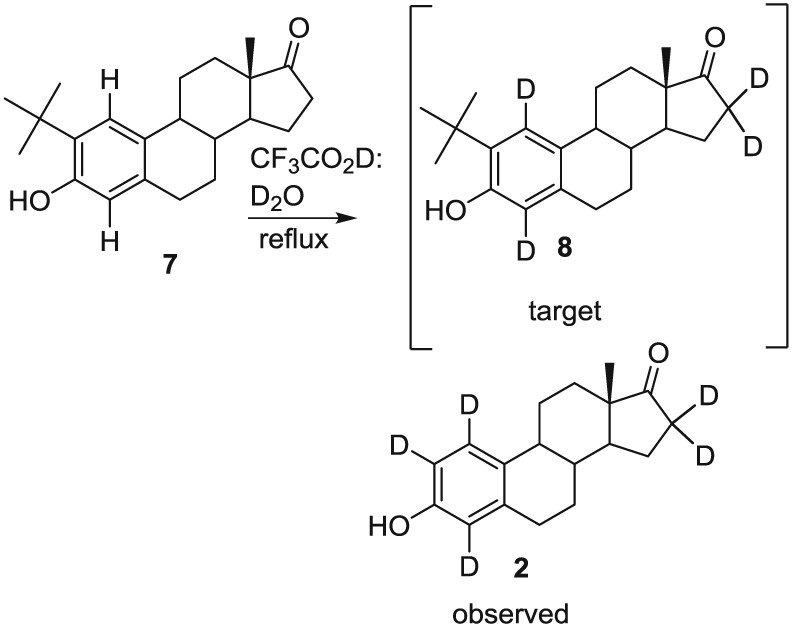
Attempted synthesis of 2‐*t*‐butylestrone‐1,4‐*d*_2_

Although the phenol functional group makes exchange at positions 2 and 4 of the estrone facile, exchange at position 1 does not occur without the presence of *t*‐butyl alcohol. The role of *t*‐butyl alcohol in affecting exchange at position 1 was discovered by an unsuccessful synthetic approach to estrone‐1‐*d*, where 2‐*t*‐butylestrone was subjected to acid‐catalyzed exchange in hopes of producing 2‐*t*‐butylestrone‐1,4,16α,16β‐*d*_*4*_ (**8**), but instead estrone‐1,2,4,16α,16β‐*d*_5_ was isolated (**2,** Figure 2). The loss of the *t*‐butyl and concurrent exchange at position 1, although unexpected, leads to the idea that back exchange of protons 2 and 4 could be realized using acid conditions without *t*‐butyl alcohol. The faster exchange of the phenolic‐activated protons in comparison with the protons at position 16 allows for the production of either **3** or **4** (Figure 1).

The exchange recipe developed was conducted in a mixture of deuterated solvents.^1^ Observation by nuclear magnetic resonance (NMR) could be done directly on the reaction mixture to follow the course of exchange to reveal the role of the *t*‐butyl catalyst. This study presents the NMR spectroscopy of both 2‐*t*‐butylestrone exchange and exchange with estrone in the presence of *t*‐butyl alcohol to produce the estrone product **2**. The NMR results and the proposed mechanistic details could provide insight into the labeling of other arene substrates at unactivated locations.

## 2 Experimental

NMR spectra were obtained on a Bruker 400‐MHz Avance III spectrometer using a 5‐mm double resonance broad band probe (model BBO). Estrone was purchased from Steraloids, Inc. (Newport, RI) and used as received, and a proton NMR is included in the Supporting information. 2‐*t*‐Butylestrone (**7**) was made by using the method of Liu et al.^12^ 2‐(*t*‐butyl‐*d9*)estrone was made by the same method by using *t‐*butyl alcohol‐*d10* (98%, Cambridge Isotopes, Andover, MA). Proton and carbon spectra for both 2‐*t*‐butylestrone and 2‐(*t*‐butyl‐*d9*)estrone are included in the Supporting information. All other chemicals were obtain from Fisher Scientific Co. (Fair Lawn, NJ) or Aldrich Chemical Co. (Milwaukee, WI) and used as received.

### 2.1 NMR analysis of *t*‐butylestrone and estrone exchange reactions

Trifluoroacetic anhydride (19.4 g, 92.5 mmol) was placed into an oven‐dried 50‐mL round bottom flask, open to air, and equipped with an anhydrous magnesium sulfate drying tube. The flask was cooled to 0°C and then carefully charged with D_2_O (99%, Cambridge Isotopes) dropwise (3.70 g, 185 mmol). 2‐*t*‐Butylestrone (**7**) (0.250 g, 0.765 mmol) was added at room temperature (RT) and stirred for 30 min before removal of an NMR aliquot, approximately 750 μL, using an oven‐dried glass pipette. NMR analysis was performed directly on the deuterated solvent system locking the instrument to D_2_O. NMR analysis was done at RT collecting 16 scans. After attachment of a reflux adapter, the solution was gently refluxed (75°C), and NMR aliquots were removed at 1, 2.5, 5, and 22 h using oven‐dried pipettes and NMR tubes, allowing the solution to cool to RT before removal of each aliquot.

The exchange of estrone was done in a similar manner by adding estrone (**1**) (0.200 g, 0.740 mmol) and *t*‐butyl alcohol (55 mg, 0.74 mmol) in lieu of 2‐*t*‐butylestrone.

Reactions done *without* sample removal typically produced **2** with 90% to 92% of H1, H2, and H4 exchanged as determined by NMR integration.^1^ Back exchange of **2** to produce **4** resulted in 87% deuterium labeling at C1.^1^

### 2.2 Thermodynamic calculations of abbreviated σ‐complexes

Structures were generated using Gaussview software (Gaussian, Wallingford, CT), minimized via molecular mechanics (MM2), and the resulting structures were used as starting geometries for density functional calculations using Gaussian 09 software^13^ (Gaussian, Wallingford, CT). Geometries and thermal correction to the free energy were calculated using the M06‐2X functional of Zhao and Truhlar^14^ using Dunning's triple‐zeta, correlation‐consistent, polarized basis set, cc‐pVTZ.^15^ The resulting geometries were used to calculate the electronic energy at a higher level quadruple‐zeta basis set, cc‐pVQZ, also using the M06‐2X functional. The thermal corrections from the lower‐level geometry calculations were used to calculate the total free energy at the M06‐2×/cc‐pVQZ level of theory. The modeling nomenclature for this method is M06‐2X/cc‐pVQT//M06‐2X/cc‐pVTZ. The effects of solvation were included in the geometry optimization by use of the polarizable continuum model (PCM).^16^ Gaussian route section and energies included in Supporting information.

## 3 Results and Discussion

### 3.1 NMR analysis of deuterium exchange with 2‐*t*‐butylestrone

**Figure 3:**
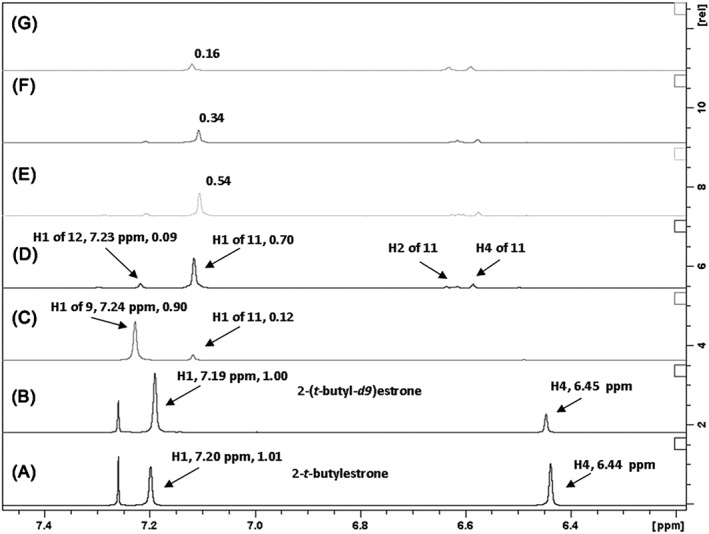
All spectra have the H1 integration value annotated as calibrated by the 3H singlet at C18. A, Proton spectrum of 2‐*t*‐butylestrone, 7, in CDCI_3_. B, Proton spectrum of 2‐(*t*‐butyl‐*d9*)estrone in CDCI_3_. C, Spectrum of 2‐*t*‐butylestrone in 50:50 CF_3_CO_2_D : D_2_O at RT for 30 min. D–G, Spectrum in of 2‐*t*‐butylestrone 50:50 CF_3_CO_2_D : D_2_O at reflux 1, 2.5, 5, and 22 h, respectively

Figure 3 shows the proton NMR spectra (arene region) of the starting 2‐*t*‐butylestrone (**7**), 2‐(*t*‐butyl‐*d9*)estrone, and the results of mixing 2‐*t*‐butylestrone in 50:50 CF_3_CO_2_D: D_2_O at various times and temperatures. To determine the nature of the *t‐*butyl group, which becomes transparent in the proton NMR after reflux (*vida infra)*, 2‐(*t‐*butyl‐*9d*)estrone was synthesized in the same manner as unlabeled **7** using *t*‐butyl alcohol‐*d10*. The chemical shift of H1 for labeled **7** is slightly upfield from unlabeled **7**, 7.19 and 7.20 ppm, respectively, (Figures 3A and 3B). This upfield shift, although small, will help track the fate of the *t*‐butyl group as the exchange proceeds. Figure 3 also has the integration value of H1 listed in each spectrum to indicate the extent of exchange. The 3H singlet at C18 was used to calibrate the integration because no change occurs at this remote location.

**Figure 4:**
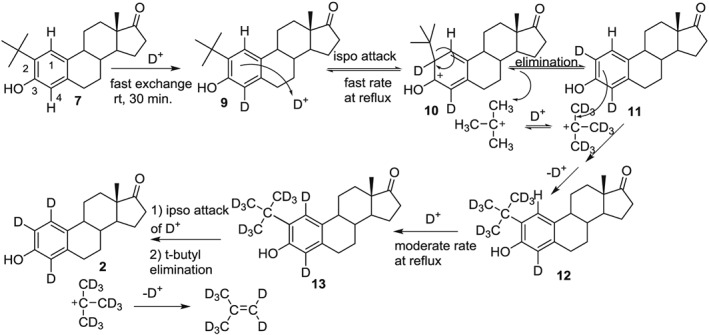
Proposed mechanism on the conversion of 7 to 2

**Figure 5:**
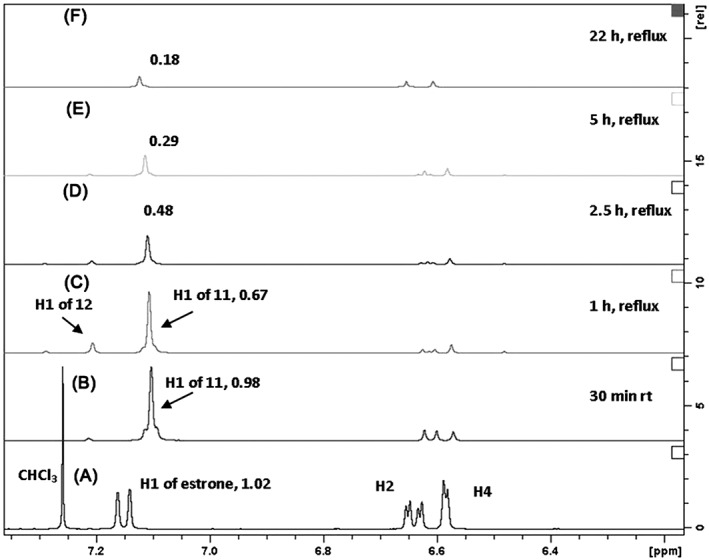
All spectra have the H1 integration value annotated as calibrated by the 3H singlet at C18. A, Proton spectrum of estrone, 1, in CDCI_3_. B, Spectrum of estrone and 1.0 Eq of *t*‐butyl alcohol in 50:50 CF_3_CO_2_D : D_2_O after 30 min at RT. C–F, Spectrum in 50:50 CF_3_CO_2_D : D_2_O at reflux 1, 2.5, 5 and 22 h, respectively

When 2‐*t*‐butylestrone is mixed in a 50:50 solution of CF_3_CO_2_D:D_2_O at RT for 30 min, the rapid exchange of the activated proton H4 is observed while the proton H1 remains. The 9H singlet for the *t*‐butyl group of **7** is still observed (see Supporting information for fully expanded spectra) as is the proton H1, which shifts downfield to 7.24 ppm in the new solvent system (Figure 3C). After 1 h at reflux in the 50:50 CF_3_CO_2_D: D_2_O exchange solvent, H1 proton shifts upfield from 7.24 to 7.10 ppm, which is the chemical shift of H1 of estrone in the same solvent system (*vide infra*, Figure 5B). Concurrent with this upfield shift is the complete disappearance of the 9H singlet of the *t*‐butyl group in **7**. Also after 1 h of reflux (Figure 3D), a small signal is observed 0.01 ppm upfield of the signal seen Figure 3C. This upfield shift would be consistent with the replacement of an unlabeled *t*‐butyl group with that of a *t*‐butyl containing 9 deuteriums (structure **12**, Figure 4). As the reflux continues, the signal for H1 continually decreases as further exchange with deuterium occurs. The small amount of back exchange at H2 and H4 displayed consistent integration values of 0.07 each in spectra 3.D through 3.G.

Figure 4 shows a mechanism consistent with these observations (note that exchange at C16 also occurs but has been omitted for clarity). The initial exchange of H4 is rapid due to the orthoproximity of the hydroxyl group. The chemical shift observed at 7.24 ppm in Figure 3C is that of H1 with a neighboring, unlabeled, *t*‐butyl group still present. As the temperature is increased, the ipso attack of a deuteron at C2, followed by *t*‐butyl elimination, results in **11**, where the H1 chemical shift has moved upfield to 7.10 ppm (Figure 3D). The *t*‐butyl carbocation would also undergo deuterium exchange, and its reversible attack back on the arene would not be observed in the proton NMR as a 9H singlet. However, small amounts of a *t‐*butyl containing deuteriums are indicated by the small signal at 7.23 ppm, which would correspond to H1 of **12** in Figure 4. This upfield shift is consistent with synthesized standards, as shown in Figures 3A and 3B. Carbocation exchange and internal scrambling are known to occur rapidly even at −70°C^17^. When present on the arene ring, the *t*‐butyl activates the C1 position toward exchange, and the H1 proton gradually disappears from the proton NMR as it is exchanged with deuterium. Presumably, the *t*‐butyl group is lost as methylpropene‐*d8* via internal β‐elimination.

### 3.2 NMR analysis of *t*‐butyl‐catalyzed exchange of estrone

If the reversible exchange of the *t*‐butyl was indeed responsible for the exchange at C1, it should not matter if it were covalently bonded to the arene ring before reaction in the exchange solvent. To test this, we mixed estrone and 1 equivalent of *t*‐butyl alcohol under the same conditions and also generated deuterium labeled steroid **2**.^1^ Less than 1 equivalent could be used, but diminished exchange was observed around 0.3 Eq of *t*‐butyl alcohol.^1^ Figure 5 shows the proton NMR spectra of this reaction recipe at similar times to those done with 2‐*t*‐butylestrone. Again after just 30 min at RT, the activated protons H2 and H4 are rapidly exchanged. The 9H singlet of t‐butyl alcohol is observed at RT (Figure 5B), but as the solution is heated to reflux, this singlet disappears, and a small singlet at 7.23 ppm corresponding to the H1 proton of **12** appears. As the reflux continues, the H1 proton diminishes in a similar manner as that observed in Figure 3. The back exchange of H2 and H4 was observed to the same extent as in Figure 3; their signals consistently integrated to 0.07 each. When the reaction is left undisturbed, the amount exchanged at H1, H2, and H4 is greater than 90% for product **2**, as determined by NMR integration.^1^

**Figure 6:**
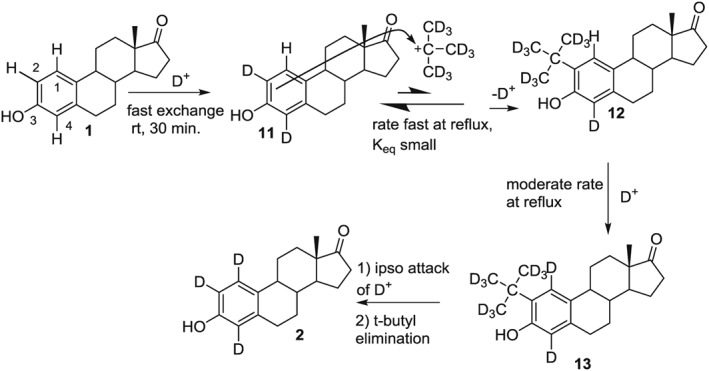
Proposed mechanism on the conversion of 7 to 2

Figure 6 shows a proposed mechanism consistent with the NMR data in Figure 5. The exchange of estrone protons H2 and H4 is rapid and as the medium is brought to reflux, and the electrophilic substitution of the *t*‐butyl occurs, activating the proton H1. Once **12** is produced, exchange and loss of the *t*‐butyl occur, as shown in Figure 4. Electrophilic aromatic substitution reactions become more reversible with relatively less reactive electrophiles, such as the *t*‐butyl cation.^18^. The result in both cases is exchanged at all 3 arene positions of the estrone A ring. Once exchange at H1 occurs, its back exchange in the absence of a *t*‐butyl catalyst is not observed. This allows selective back exchange at positions 2 and 4 with protic acids.

### 3.3 Computational analysis of 2 isomeric σ‐complexes

**Figure 7:**
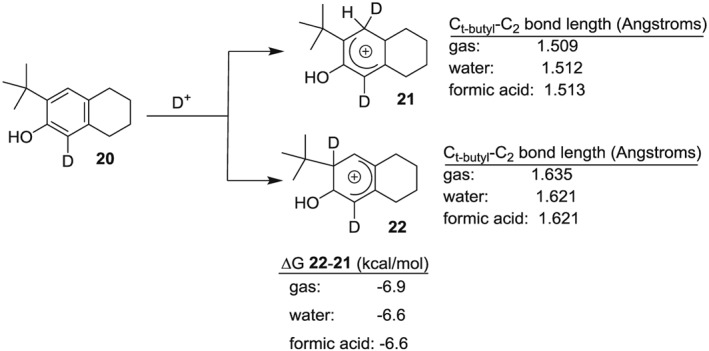
Relative energies of the 2 isomeric *σ* complexes resulting from D^+^ attack at C1 and C2

NMR analysis shows that the exchange of the *t*‐butyl group occurs faster than the exchange of the proton at C1. Ortho attack relative to the strongly activating hydroxyl group (ispo attack at C2) would be expected to produce a σ‐complex more stable than ortho attack to the less activating *t*‐butyl group (addition at C1). To gauge the relative stability of the 2 isomeric σ‐complexes resulting from attack at C1 versus C2, we modeled abbreviated structures. Figure 7 shows density functional calculations done at the MP06x/cc‐pVQZ//MP06x/cc‐pVTZ level of theory. The σ‐complex resulting in an ipso attack (**22,** Figure 7) was shown to be more stable by 6.6 kcal/mol in an aqueous and formic acid solvent system. Formic acid was modeled because a 50:50 mixture of trifluoroacetic and water would have a dielectric constant similar to formic acid. Negligible change in relative free energy was observed in going from gas phase to either solvent system.

A significant structural difference between **21** and **22** was the bond length connecting the *t*‐butyl group to C2. The ipso attack at C2 significantly lengthened the C_*t*‐butyl_‐C2 bond length from 1.509 to 1.635 Å (gas phase), indicating that the loss of the *t*‐butyl would occur quickly after an ipso attack. The results from density functional calculations are in good agreement with NMR analysis.

## 4 Conclusions

We have determined the role of *t*‐butyl alcohol in achieving deuterium exchange at the unactivated C1 position of estrone. The mechanism involves reversible addition/elimination attack of the *t*‐butyl group with concurrent slower exchange at the unactivated C1 position. In the absence of the *t*‐butyl catalyst, the labeled position at C1 is stable in acidic medium, making this label useful in investigations involving estrone and estrone metabolites where reaction at C1 occurs. This method could be extended to the labeling of other phenolic metabolites at a position not prone to electrophilic exchange.

## Supporting information

Supporting info item
