# Supplementary material for: NMR analysis of t‐butyl‐catalyzed deuterium exchange at unactivated arene localities
Source: J Labelled Comp Radiopharm. 2016 Sep 19;59(12):500–5. doi: 10.1002/jlcr.3440 (PMC5129451; doi:10.1002/jlcr.3440)
Supplement: Supplementary file 1 — Supporting info item [file JLCR-59-500-s001.docx]

**Supporting Information**

NMR Analysis of t-Butyl Catalyzed, Deuterium Exchange at Unactivated Arene Localities.

**Table of Contents**

1. Expanded NMRs from Figure 3…………………………………………………………2

2. Expanded NMRs from Figure 5…………………………………………………………3

4. Proton NMRs of 2-*t*-butylestrone and 2-(*t*-butyl-*d9*)estrone……………………………..4

4. Gaussian input files for structures **21** and **22**…………………………………………….5

5. Energies of Gaussian outputs for **21** and **22**……………………………………………..6

Expanded spectra of 2-*t*-butylestrone exchange reactions

9H singlet of *t*-butyl group no longer present at 1h reflux and after

9H singlet of *t*-butyl group

**
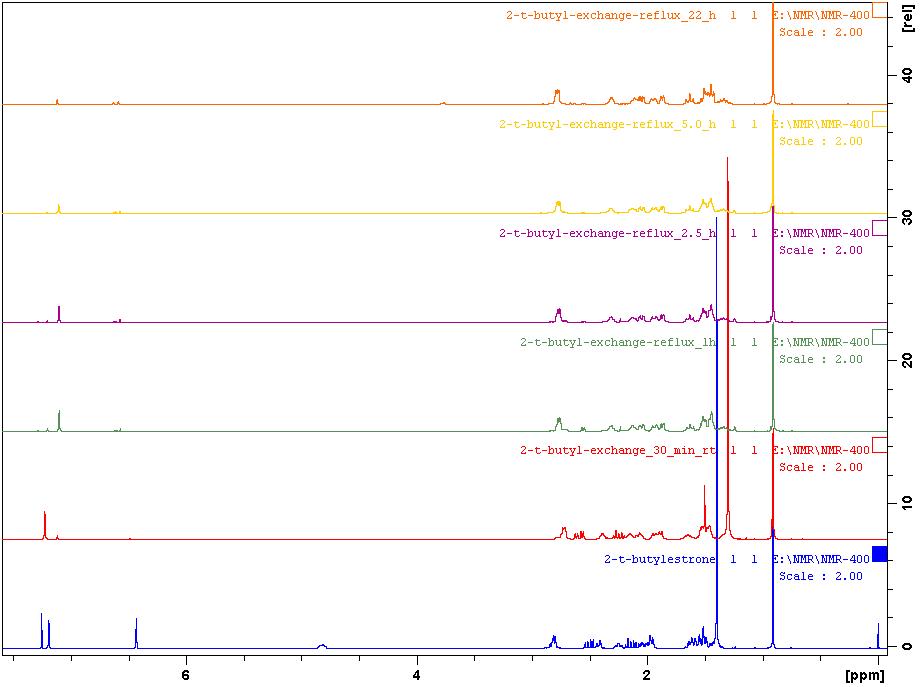
**

**F**

**E**

**D**

**C**

**A**:Proton spectrum of 2-t-butylestrone in CDCl_3_; **B**: Spectrum in 50:50 CF_3_CO_2_D : D_2_O at rt for 30 min; **C-F**: Spectrum in 50:50 CF_3_CO_2_D : D_2_O at reflux 1, 2.5 , 5 and 22 h, respectively.

**B**

**A**

**A**

Expanded spectra of *t*-butyl catalyzed, estrone exchange reactions.

9H singlet of *t*-butyl group of *t*-butyl alcohol

9H singlet of *t*-butyl group at C2 of estrone


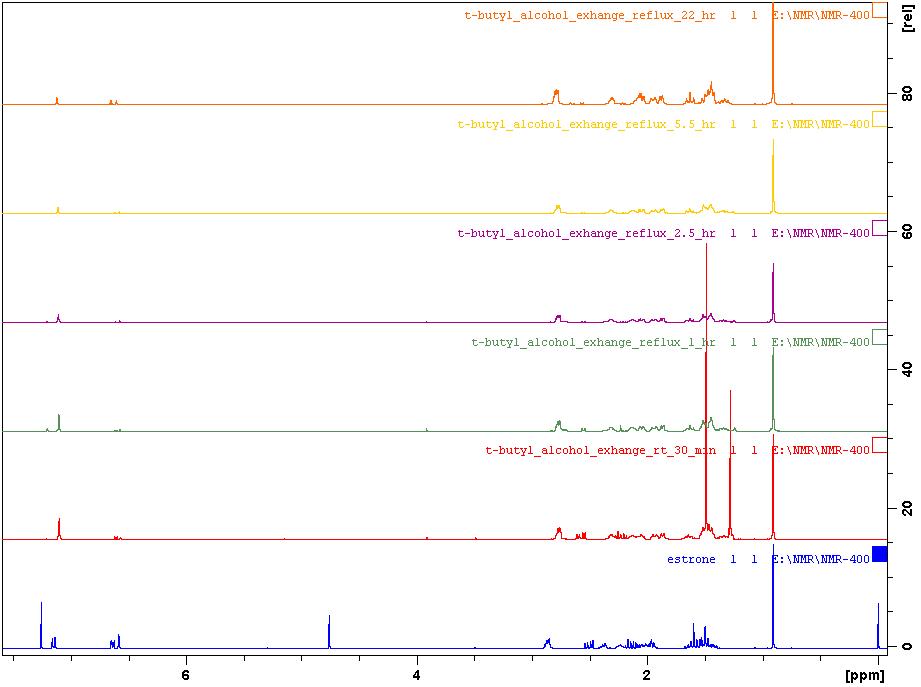


**A**

**F**

**E**

**D**

**B**

**C**

**F**

**F**

9H singlet of *t*-butyl group of t-butyl alcohol

**A:**Proton spectrum of estrone in CDCl_3_; **B**: Spectrum of estrone and 1.0 eq. of t-butyl alcohol in 50:50 CF_3_CO_2_D:D_2_O after 30 min at rt; **C-F**: Spectrum in 50:50 CF_3_CO_2_D:D_2_O at reflux 1, 2.5 , 5 and 22 h, respectively.

Proton NMR of 2-*t*-butyl estrone (top) and 2-(*t*-butyl-*d9*)estrone in (bottom). Both spectra are in CDCl_3_

t-butyl, 9H singlet absent

t-butyl, 9H singlet

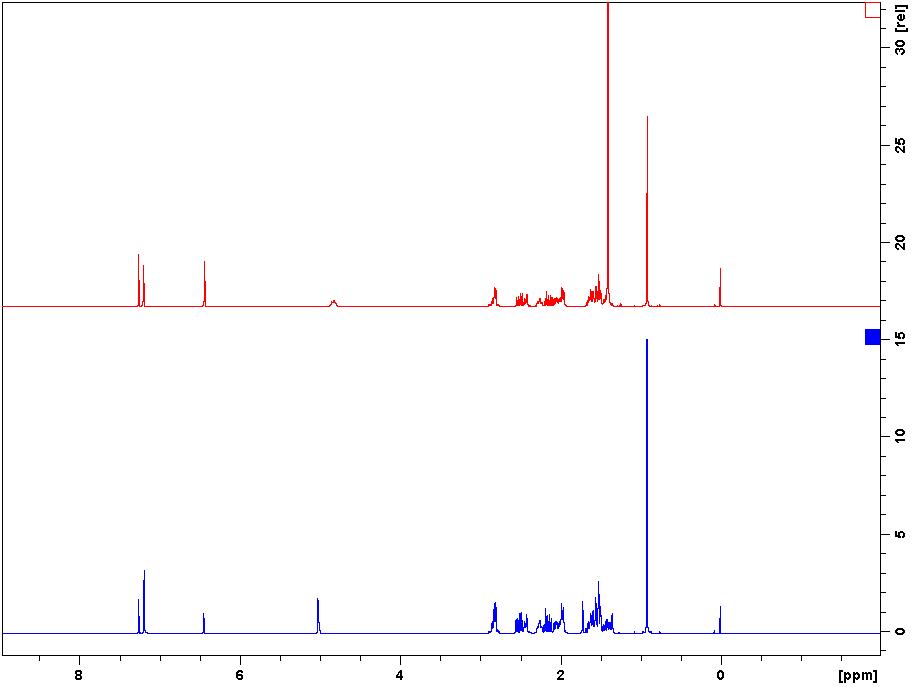


The follow input file is for a solvent system in water. For gas phase calculations, remove the

SCRF key in both route sections. For formic acid calculations, solvent=water to solvent=formic acid.

Structure 21:

%chk=D:\Stack_data\IPSO_Calc\ipso_1-water.chk

%mem=8GB

%nprocshared=8

# opt=tight freq=noraman cc-pvtz pop=npa empiricaldispersion=gd3

integral=ultrafinegrid m062x SCRF=(solvent=water)

ispo complex attack at c1, structure 21, Figure 8

1 1

C -4.98230000 0.05190000 -0.01080000

C -4.82130000 -1.26280000 -0.28830000

C -3.61070000 -1.84870000 -0.24780000

C -2.45950000 -1.24620000 0.07800000

C -2.50180000 0.24680000 0.45050000

C -3.80370000 0.94570000 0.32770000

O -5.87240000 -2.05700000 -0.64180000

C -6.34810000 0.76340000 -0.10530000

C -7.46070000 0.00960000 0.66010000

C -6.34260000 2.19520000 0.48970000

C -6.73070000 0.89410000 -1.59540000

C -1.14690000 -1.98570000 0.12120000

C 0.04310000 -1.04000000 -0.08670000

C -0.02810000 0.14250000 0.88850000

C -1.24310000 1.03020000 0.58990000

H -3.97120000 1.44190000 1.31070000

H(Iso=2) -3.69730000 1.71380000 -0.47320000

H(Iso=2) -3.55920000 -2.92320000 -0.49610000

H -5.55470000 -2.93430000 -0.91020000

H -8.39630000 0.61040000 0.71820000

H -7.77310000 -0.94080000 0.18070000

H -7.14260000 -0.21390000 1.70390000

H -7.33780000 2.68430000 0.38650000

H -5.62860000 2.87470000 -0.02560000

H -6.10940000 2.19020000 1.57840000

H -6.83400000 -0.09330000 -2.09610000

H -7.70410000 1.42000000 -1.72080000

H -5.96090000 1.47160000 -2.15630000

H -1.06670000 -2.47280000 1.12190000

H -1.12210000 -2.79160000 -0.64940000

H 0.99630000 -1.60190000 0.06300000

H 0.04760000 -0.65820000 -1.13550000

H 0.90420000 0.75270000 0.81480000

H -0.08460000 -0.24030000 1.93540000

H -1.36550000 1.78620000 1.40060000

H -1.07770000 1.58200000 -0.36560000

--Link1--

%chk=D:\Stack_data\IPSO_Calc\ipso_1-water.chk

%mem=8GB

%nprocshared=8

# integral=ultrafinegrid m062x cc-pvqz empiricaldispersion=gd3 Geom=allcheck guess=read SCRF=(solvent=water)

Structure 22:

%chk=D:\Stack_data\IPSO_Calc\ipso_2-water.chk

%mem=4GB

%nprocshared=8

# opt=tight freq=noraman cc-pvtz pop=npa

empiricaldispersion=gd3 integral=ultrafinegrid m062x SCRF=(solvent=water)

ispo complex attack at c2, structure 22, Figure 8

1 1

C 0 -5.4892 0.1200 0.4885

C 0 -5.2458 -1.3412 0.3767

C 0 -3.8063 -1.8224 0.1211

C 0 -2.7621 -1.0184 0.3793

C 0 -2.9636 0.2421 0.8164

C 0 -4.1969 0.7773 0.9063

O 0 -6.1707 -2.2859 0.8695

C 0 -6.0837 0.7135 -0.8152

C 0 -7.3907 -0.0246 -1.1812

C 0 -5.0960 0.5845 -1.9937

C 0 -6.4250 2.2075 -0.6193

C 0 -1.3734 -1.5946 0.1911

C 0 -0.2654 -0.5372 0.1811

C 0 -0.4723 0.3953 1.3756

C 0 -1.7998 1.1384 1.1952

H 0 -4.2892 1.8144 1.2720

H(iso=2) 0 -3.6500 -2.8552 -0.2277

H(iso=2) 0 -6.2165 0.2708 1.3233

H 0 -5.7273 -3.0672 1.1487

H 0 -7.8752 0.4248 -2.0773

H 0 -8.1268 0.0186 -0.3468

H 0 -7.2110 -1.0962 -1.4207

H 0 -5.5255 1.0134 -2.9272

H 0 -4.1427 1.1230 -1.7943

H 0 -4.8455 -0.4783 -2.2080

H 0 -5.5168 2.8259 -0.4465

H 0 -6.9285 2.6302 -1.5180

H 0 -7.1094 2.3569 0.2462

H 0 -1.1911 -2.3122 1.0269

H 0 -1.3234 -2.1735 -0.7619

H 0 0.7363 -1.0265 0.2305

H 0 -0.3020 0.0516 -0.7664

H 0 0.3696 1.1232 1.4581

H 0 -0.4890 -0.2010 2.3189

H 0 -2.0273 1.6895 2.1389

H 0 -1.6870 1.8983 0.3846

--Link1--

%chk=D:\Stack_data\IPSO_Calc\ipso_2-water.chk

%mem=4GB

%nprocshared=16

# integral=ultrafinegrid m062x cc-pvqz empiricaldispersion=gd3 Geom=allcheck guess=read SCRF=(solvent=water)

Summary of Energies from Gaussian Calculations:

|  | SCF | thermo_correction | G | rel. G kcal/mol |
| --- | --- | --- | --- | --- |
|  | m062x/cc-pvqz | M062x/cc-pvtz |  |  |
| SC_21_gas | -621.088014 | 0.274547 | -620.813467 | 6.9 |
| SC_22_gas | -621.101224 | 0.276716 | -620.824508 | 0.0 |
|  |  |  |  |  |
| SC_21_water | -621.156528 | 0.274874 | -620.881654 | 6.6 |
| SC_22_water | -621.168735 | 0.276565 | -620.892170 | 0.0 |
|  |  |  |  |  |
| SC_21_formic_acid | -621.156009 | 0.274919 | -620.881090 | 6.6 |
| SC_22_formic_acid | -621.168234 | 0.276578 | -620.891656 | 0.0 |
